# Symptom and Illness Experience for English and Spanish-Speaking Children with Advanced Cancer: Child and Parent Perspective

**DOI:** 10.3390/children8080657

**Published:** 2021-07-29

**Authors:** Donna S. Zhukovsky, Cathy L. Rozmus, Rhonda Robert, Eduardo Bruera, Robert J. Wells, Marlene Z. Cohen

**Affiliations:** 1 Department of Palliative, Rehabilitation and Integrative Medicine, The University of Texas M.D. Anderson Cancer Center, Houston, TX 77030, USA; ebruera@mdanderson.org; 2 Department of Pediatrics, The University of Texas M.D. Anderson Cancer Center, Houston, TX 77030, USA; rrobert@mdanderson.org (R.R.); phantomrider47@gmail.com (R.J.W.); 3 School of Nursing, The University of Texas Health Science Center at Houston, Houston, TX 77030, USA; Cathy.L.Rozmus@uth.tmc.edu; 4 VA Nebraska Western Iowa Health Care System, College of Nursing, The University of Nebraska Medical Center, Omaha, NE 68144, USA; mzcohen@unmc.edu

**Keywords:** caregiver, caregiver experience, child, cancer, patient experience, symptoms, pediatrics

## Abstract

Understanding the symptom and illness experience of children with advanced cancer facilitates quality care; yet, obtaining this understanding is complicated by the child’s developmental level and physical and psychological health factors that affect communication. The purpose of this study was to describe the symptom and illness experience of English- and Spanish-speaking children with advanced cancer as described by the child and parent. We conducted hermeneutic phenomenological, descriptive, and interpretive interviews with eligible children and parents. The interdisciplinary research team analyzed transcripts hermeneutically until consensus on theme labels was reached. Four themes and associated subthemes were identified from the interviews of the 10 child–parent dyads: 1. symptoms disrupt life (path to diagnosis, life is disrupted), 2. isolation (lack of understanding, family separations/relationships), 3. protection, and 4. death is not for children. Children and parents readily described the impact symptoms and cancer treatment had on their lives and relationships. These findings underscore the salient aspects of daily life disrupted by cancer. With a deeper understanding of symptom burden and its interference, relationship and communication implications, and anticipatory grief, the treating team may better optimize care for children and their families living with advanced cancer.

## 1. Introduction

The symptom and illness experience for pediatric patients with advanced cancer is an integral component of comprehensive cancer care. Suffering caused by symptoms such as nausea, vomiting, and fatigue, as well as symptoms limiting activities due to limb or central nervous system dysfunction, is common in children under active treatment or supportive care for advanced cancer. Concerns about eventual outcome of the cancer and its treatment cause additional suffering for both patients and their families.

Communication of these health-related concerns is complicated by the child’s developmental level, physical and psychological health status, current distress level, and patient–caregiver relationships. Consequently, pediatric clinicians may need to rely on various resources and skills, as well as collateral information, to inform their interventions.

Proxy assessments are often garnered from parents and treating clinicians, aiming to complement the child’s self-report. Agreement between reporters may vary depending on factors such as the particular symptom domain assessed, the individual rater, and patient–proxy relationship characteristics [1,2,3]. Raters may define symptoms differently. For example, investigators studying fatigue found that children, adolescents, parents, and staff [4] each described fatigue differently. Most studies evaluating symptoms in children with cancer rely on proxy report; few studies include patient reported outcomes [5,6,7].

The context in which the symptom occurs may also be relevant. The meaning of an individual symptom and its impact on daily life may vary depending upon other symptoms occurring simultaneously. Culture may impact symptom expression [8,9]. Few studies evaluating symptoms have been conducted in Hispanic children with cancer [10]. As our patient population included a substantial number of Hispanic individuals, we included Spanish-speaking cohorts as a proxy marker for ethnicity. The objective of this study was to describe the child’s symptom and illness experience, as reported by English- and Spanish-speaking children and parents. With enhanced knowledge of the experience, the treating team may better guide assessment, communication, and medical and other care interventions.

## 2. Methods

### 2.1. Participants and Procedures

Study participants were recruited as part of a larger study designed to characterize the symptoms of pediatric oncology outpatients over the previous two days and to evaluate the association between child reports and proxy ratings [11]. Eligible children were ages seven to eighteen years of age, had advanced cancer (defined as relapsed, metastatic, or progressive), were established patients of the Division of Pediatrics at one National Cancer Institute-designated comprehensive cancer center, spoke and understood English or Spanish, and had an available family caregiver who spoke the same language. Eligible family caregivers were the parent or legal guardian of an eligible child. Children and family caregivers were eligible only if both members of the dyad agreed to participate. Patients and family caregivers with severe cognitive impairment, psychiatric disturbance, or an unstable medical condition that would preclude data collection or add to participant burden were ineligible.

Once participant consent or assent with parental permission, as age appropriate, was obtained for participation in the main study, children and their family caregivers were invited to participate in the interview portion of the study until data were saturated. Interviews, conducted by the bilingual research coordinator, took place separately, but as close together in time as possible, so that patients and their caregivers did not discuss interviews with each other before both were completed. Interviews were conducted face-to-face in the outpatient clinic. On study conclusion, family caregivers were reimbursed for parking or transportation expenses and the child received a $10.00 gift card.

### 2.2. Data Collection

The children’s demographic and clinical information were collected from the electronic medical record and by child report. Family caregivers provided their own demographic information.

Hermeneutic phenomenological, descriptive, and interpretive research methods guided the study [12]. These research methods are used to determine how people interpret their lives and make meaning of what they experience. The purpose is to understand the meaning of an experience from the perspective of the persons who have had that experience [12].

**Figure 1:**
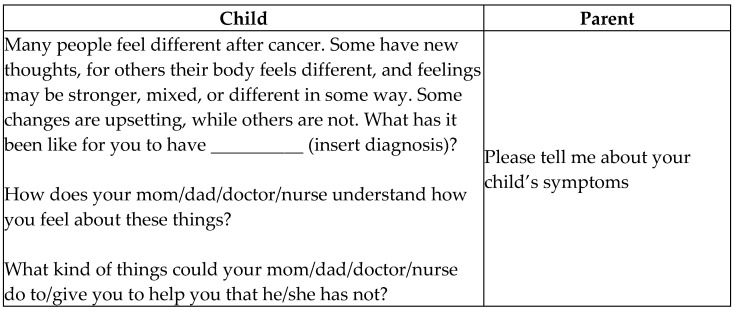
Interview questions.

Children were asked to describe their experience with their diagnosis and symptoms. Caregivers were asked to describe their child’s symptoms. The interview questions are detailed in Figure 1. Open-ended questions were used to ensure that the person being interviewed, rather than the interviewer, determined the content. Patients and their caregivers were asked for specific examples to illustrate general statements and encouraged to describe their experiences fully. The goal was to help them clarify their own meaning and to ensure that the meaning of their experiences was clearly understood.

### 2.3. Analysis

Analysis of demographic data was descriptive, with medians and percentages, as appropriate. For the interviews, as is usual with hermeneutic phenomenological designs [12], analysis began during data collection. Interviews were audio-taped and transcribed verbatim, with transcription accuracy verified by a member of the research team. Interviews conducted in Spanish were transcribed verbatim and then translated into English. Spanish–English translations were verified by a second bilingual research coordinator and differences resolved by consensus. Phenomenological analysis was performed by three investigators (MZC, CLR, and DSZ) who initially read each transcript several times to get a sense of each interview as a whole. Themes in each transcript were examined line by line, underlining and labelling passages with tentative theme labels. Passages from interview text and labels, or theme labels, for each interview were compared with passages and themes among and between all other interviews with both patients and family caregivers, including comparison of themes discussed by each ethnic group [12].

Procedures to ensure scientific rigor included having the analysis conducted by three researchers. Theme labels and passages were reviewed until consensus was reached. While verification of the theme labels with patients and parents would have been ideal, half of the patients were no longer alive by the time data analysis was completed. Interviews were conducted until no new themes emerged and data saturation was reached, meeting the standard criterion used in qualitative research [12].

## 3. Results

Interviews occurred with 10 children and 10 family caregivers. All caregivers were the child’s parent. Clinical and demographic data for the ten interviewed children and their family caregivers are displayed in Table 1 and Table 2, respectively. Chemotherapy was in progress for five children and planned for an additional two. Nine of the 10 children were symptomatic within the past 2 days. Median number (range) of child-reported symptoms was seven (one–nine) for the 11–18 year-old group and four (one–five) for the 7–10 year-old group. Fatigue, pain, itch, and nausea were among the most common symptoms [11].

### 3.1. Phenomenological Themes

Four themes were identified from the interviews of the children, adolescents, and their parents: 1. *symptoms disrupt life,* 2. *isolation*, 3. *protection*, and 4. *death is not for children*. While descriptions of content were the same for children, adolescents, and their parents, children’s descriptions differed by their developmental level and sometimes were affected by their diagnosis. Younger children gave shorter, more concrete descriptions than adolescents and parents. Notably, similar themes were found regardless of participant ethnicity or language of interview.

### 3.2. Symptoms Disrupt Life

#### Path to Diagnosis

Parents frequently described symptoms as a path to diagnosis. The initial symptoms led parents to believe that something was wrong with their child, thus beginning the path to cancer diagnosis. The mother of a 9-year-old daughter noted,

“She would wake up in the middle of the night and she would cry, she would scream, she would say her arm hurt, her leg hurt, she was tired, she slept all the time and she just wasn’t herself. And you know, and then you are telling them look, a child that wants attention doesn’t wake up at one o’clock in the morning screaming at the top of their lungs, that they are hurting. There is something wrong with them.”

The mother of a 10-year-old boy noted,

“You know he started losing weight, appetite, which we just thought that was, he was a picky eater, he didn’t want to eat, but, you know, we look back now and we think, well, that was a sign; we just kind of ignored that … we didn’t realize that was one of the symptoms until we looked back …”

In some cases, symptoms were described primarily as the past path to diagnosis, with few current symptoms. For others, symptoms that had led to diagnosis had reoccurred and/or were ongoing.

### 3.3. Life Is Disrupted

Life is disrupted since the cancer diagnosis described the presence of physical symptoms and emotional distress that limited activity, which then created more distress. However, it was not just the symptoms but the impact these symptoms had on the child that made life different.

The mother of a ten-year-old boy said:

“OK, his major symptom is pain, obviously. Umm … back pain was his major symptom and leg pain … the point of that is it limited his walking, limited his activity … limited his playing with other kids … so it limited everything, really.”

Symptoms and their meaning in terms of the illnesses were discussed by parents who noted, for example, that their child could not “carry on a normal life” because of incapacitating symptoms or that their child would not be able to attend college.

Life was also disrupted by psychological symptoms. A 12-year-old girl poignantly described the distress linked with her symptoms as:

“Well pain, as in headaches, no. But pain, as in of the heart.”

Life disruptions included major losses and associated psychological symptoms. The mother of a 15-year-old boy said:

“It is just that he gets angry because he thinks … we overprotect him, right? But at the same time he gets sad because he wants to, like, for example … he was there at school, he likes American football a lot and he cannot [play].”

The children and their parents described the child as feeling different and no longer normal. A 15-year-old boy noted he felt,

“Bad, sad. Because I cannot do anything … Well, like a normal child.” He also said, “Sometimes I am not myself; I am not the same person.”

### 3.4. Isolation

#### 3.4.1. Lack of Understanding

Children and parents expressed that other people may not understand the child and what the child is going through, leaving the child to feel alone and isolated. The mother of a 10-year-old boy remarked, “… Now he can probably go to school, some, but he doesn’t want to, because now he thinks he’s labeled again…”

#### 3.4.2. Family Separations/Relationships

Families were forced to endure separations from family and friends, which affected relationships.

A 9-year-old girl noted,

“Because he [dad] is always home trying to take care of my sister, and my momma has to stay out here with me so she can like be here to tell the doctor all the information cause dad’s home working trying to make money … I wish he would like come out here and stay with us until December, but he can’t cause he’s working and has to take care of my sister because she’s going to school.”

The unpredictable nature of the child’s course also impacted family relationships. The 42-year-old mother of a 15-year-old son commented that,

“He (brother) never knows who’s coming to get him from school, like today … I said we may or may not be home.”

These separations were particularly important because of the importance of support of and for family and friends. Parents, children, and adolescents each compensated by optimizing the available support. One 9-year-old girl stated: “I try to help out if my mom is upset or if anyone is upset, I like try to help them feel better.” She also described caring for other children who are sick: “I try to care about other people, help a lot of people and try to give them spirit to not give up.”

### 3.5. Protection

Potential for harm led to various ways to try to protect children. The mother of a 9-year-old son who was physically active prior to leukemia noted:

“I am scared about him. They told me that he had hurt his backbone, fractured-so to also not let him run because one fall or something would hit him badly-harm him.”

Another mother reflected on how she protected her son by limiting information.

“Well, he took it a little bad … he does not know how severe it is … because I have also not wanted to tell him because, well … the more sick [sic] he is going to get or get sad; I did not want that. He hardly asks anything.”

Children limited information disclosure to parents, possibly as a means of protecting their sense of normalcy and perhaps as a means of protecting their parents. The mother of a 10-year-old boy noted,

“I think he hides a lot. … He didn’t tell us how, how bad he was hurting cause [sic] he didn’t want to feel different, or to look at him different.”

### 3.6. Death Is Not for Children

Finally, death is not for children reflects some parents’ fear and reluctance to discuss the diagnosis or prognosis with their child, although some of the children were clearly aware that they could die.

The mother of a 9-year-old daughter said:

“Well, when she started asking me questions about dying … You know, she could die from this. Just the thought of her, I mean, she didn’t want to die. You know, she knows, ‘I’m going to heaven, I know that, but I don’t want to die.’ Just stuff like that … You never want to try and take any kind of hope or anything away from them.”

In a few cases, parents did not allow the child or adolescent to know their diagnosis. The mother of a 12-year-old daughter stated:

“But she does not know, she does not know with full knowledge. Why? Because I think that I, as a mother, see that knowing certain things at times, instead of helping us, it can lead us to like “Why should I continue, why should I fight?’ Because like, what I feel like I am limiting her …”

The discussion of death was primarily from parents. A mother noted that one of her 15-year-old son’s “new friends” from the hospital died two weeks before this interview, although the son did not mention this friend when interviewed. Despite parents’ attempts to protect their children from thoughts of death, some children discussed their awareness of death and prognosis. One 17-year-old boy described being unable to finish the year in high school, not seeing his friends, and added,

“It’s either this or a blunt way to put it, it is six feet under.”

## 4. Discussion

Although specifically asked about symptoms, patients and parents focused primarily on the illness experience rather than the symptoms themselves. Potential reasons for this are multiple and bear further study. Some children and parents might believe that treatment-related symptoms, since transient, need to be tolerated, while others might believe symptoms were immutable, given their limited experience with aggressive symptom management [11]. Others might believe that a focus on symptoms would distract the oncology team from managing the cancer. Regardless, persistent symptoms, irrespective of symptom severity, potentially serve as pervasive reminders of the cancer diagnosis and how it has changed their lives, leading to a focus on the illness experience. Alternatively, children’s and parents’ choice to focus on the illness experience may reflect their experiences with advanced cancer in the treatment environment (their family and families of other patients with advanced cancer) and accordingly, dominated the information shared. Furthermore, throughout the interviews, their focus on relationships, interactions, and thoughts about the future were among the most salient aspects to their conversations. Thus, the themes (*symptoms disrupt life*, *isolation, protection,* and *a fear and belief that death is not for children)* reinforce the scope of health care needed for symptom management and might suggest related structured clinical interview assessment domains (see Table 3).

Notably, similar themes were found among participants, regardless of ethnicity or language of interview, although data were limited due to the small number of participants. However, behavior and beliefs, identity, daily life, coping skills, one’s life story, values, world view, and confrontation with death were all touched by illness and its symptoms.

The broad impact of symptoms beyond the physical has been noted by another investigator who has coined the term “feeling states” to better represent the impact of cancer and cancer symptoms on children and adolescents [13,14]. Our findings similarly suggest that for children, evaluation of individual symptoms distinct from accompanying symptoms or by contributing dimensions such as intensity, might be of more limited value than for adults. Symptoms may instead serve as a proxy for the symptom experience and its impact on the child–family unit. Prospective studies of these important preliminary findings are expected to guide the development of future studies that better characterize symptom expression, its impact of daily living and response to interventions. This information would likely facilitate more optimal tailoring of support to patients’ and families’ needs. Such studies may also lead to the development of different tools to assess the overall symptom experience for pediatric patients and their families.

Relationships were focal to the illness experience. Study participants described both negative and beneficial impacts, suggesting an opportunity for professional assessment and intervention. Giving and receiving support from family and friends were described as important coping elements and were psychologically beneficial. Bereaved parents of children who have died of cancer have similarly reported psychological benefit from reciprocated social support [15]. Clearly, understanding the impact of cancer and its treatment on relationships is relevant to quality care. Furthermore, enhancing altruistic opportunities may be a powerful tool for enhancing one’s own well-being, as well as that of others, and warrants further investigation. Clinicians may be able to enhance families’ ability to cope during long separations from family and friends by linking them to support systems within the healthcare environment; programmatically, optimizing accessibility of social support may encourage clinicians to provide such support.

Communication challenges were noteworthy. Several studies have documented a difference in symptom reports between parent proxy ratings and the child’s self-report [11,16,17] of the child’s symptom between parents and their children. One contributing factor may be related to the desire to protect loved ones. Both the patients and the parents wished to protect the other and sometimes, themselves. Their tendency to withhold information highlighted an opportunity for therapeutic intervention. Talking about illness and death can be psychologically threatening. Yet, social science evidence that disclosure over time that is sensitive to the child’s developmental needs is associated with decreased anxiety and an improved sense of support [18]. Psychoeducation and subsequent guidance from healthcare team members might bridge this communication disconnect between patients and parents.

Children and adolescents described withholding information about their symptoms from their parents, essential information needed for treating symptoms. Withholding information from parents may contribute to discrepancies between caregiver proxy reports and child and adolescent self-reports of symptoms [1,2,3,11]. Discussions with children and adolescents on the importance of symptom information for their treatment and routine assessment with validated symptom assessment tools may lead to more accurate symptom reporting by children and adolescents.

Fear and belief that death is not for children was discussed exclusively by parents, despite the finding that some children demonstrated death awareness. For children with a potentially terminal illness, incomplete communication may lead to unnecessary fears and/or missed opportunities for therapeutic interventions. This “need to protect” or rescue is a fundamental human behavior for families. However, neither parents nor children are capable of protecting the other from disease progression, death, or symptoms. Moreover, parents and children discussed anticipatory grief. This finding suggests that initiating conversations related to anticipated losses and the possibility of death, though difficult, may provide psychological relief.

Data limitations include the small number of informants from only one healthcare site, the predominance of male patients and female caregivers, and the relatively high educational and income levels of parents. These factors may limit the applicability of these findings, although the themes were common to all 10 dyads.

A number of strengths are also important. Children had diagnoses that were typical of the pediatric population. Both English- and Spanish-speaking participants were included, allowing people to express themselves in their first language. Twelve of the 20 interviewed were Hispanic. Finally, the rigor of the study methodology was high, using established standards, including translation and back-translation of the Spanish interviews and validation of the analyses by the research team.

The perspective of patients and parents is important to guide assessment, discussions, and treatment. A potential semi-structured interview guide is provided in Table 3 and would be relevant for both patient and proxy interviews. To date, studies have varied in the frequency of agreement among family and professional proxy raters of the child’s symptoms [1,2]. Studies of pediatric and adult patients with cancer have suggested that patients and their families want their symptoms acknowledged, and want information about their symptoms and potential interventions [15,19]. Better understanding by healthcare professionals of the perspectives of patients and their families, including their fears and potentially detrimental strategies used to protect each other from distress, has the potential to lead to improved symptom management, greater patient satisfaction with care, and support more effective coping mechanisms.

## 5. Conclusions

Insights offered by these children and parents highlight the importance of effective symptom assessment and control, as well as the broader understanding of cancer and its impact on children and their families. Potential reasons for differences in symptom reports between children and their parents have been elucidated. Often these reasons reflect wanting to protect one another or themselves and to maintain some degree of normalcy. Further research is needed to develop the elucidated themes and associated interventions to improve the symptom and illness experience for children and their families living with advanced cancer.

## Figures and Tables

**Table 1:** Child demographics (N = 10).

Median Years of Age (Range)	11 (7–17)
Gender: Male/Female	7/3
Race: White/Hispanic	4/6
Language of interview: English/Spanish	7/3
Primary Diagnosis	
CNS tumor	3
Leukemia	3
Lymphoma	1
Sarcoma	3
Extent of Disease	
Metastatic	5
Progressive	1
Recurrent	4

**Table 2:** Parent demographics (N = 10).

Median Years of Age (Range)	43.5 (27–52)
Gender: Male/Female	1/9
Race: White/Hispanic	4/6
Education	
9th–12th grade	2
Some college or technical	4
College graduate	2
Graduate school	2
Annual Income	
$24,999 or less	3
$25,000–$49,999	4
$50,000–$74,999	2
$100,000–$149,999	1
Marital Status	
Married	5
Single/Never married	3
Single/Lives with partner	1
Divorced	1

**Table 3:** Structured clinical assessment domains suggested by themes.

Physical and Psychological Symptoms Burden Interference
Interpersonal Implications Physical separation Loss of shared activities and understanding
Communication Problems Caregiver protecting child and self Child protecting caregiver and self
Anticipatory Grief

## Data Availability

Data are available on request from the authors and will be destroyed 2 years after publication. The data are not publicly available due to the potential to lead to recognition of the study participants.
